# Supplementary material for: Differential Allele-Specific Expression Revealed Functional Variants and Candidate Genes Related to Meat Quality Traits in B. indicus Muscle
Source: Genes (Basel). 2022 Dec 11;13(12):2336. doi: 10.3390/genes13122336 (PMC9777870; doi:10.3390/genes13122336)
Supplement: Supplementary file 1 [file genes-13-02336-s001.zip › Supplementary Data S1.pdf]

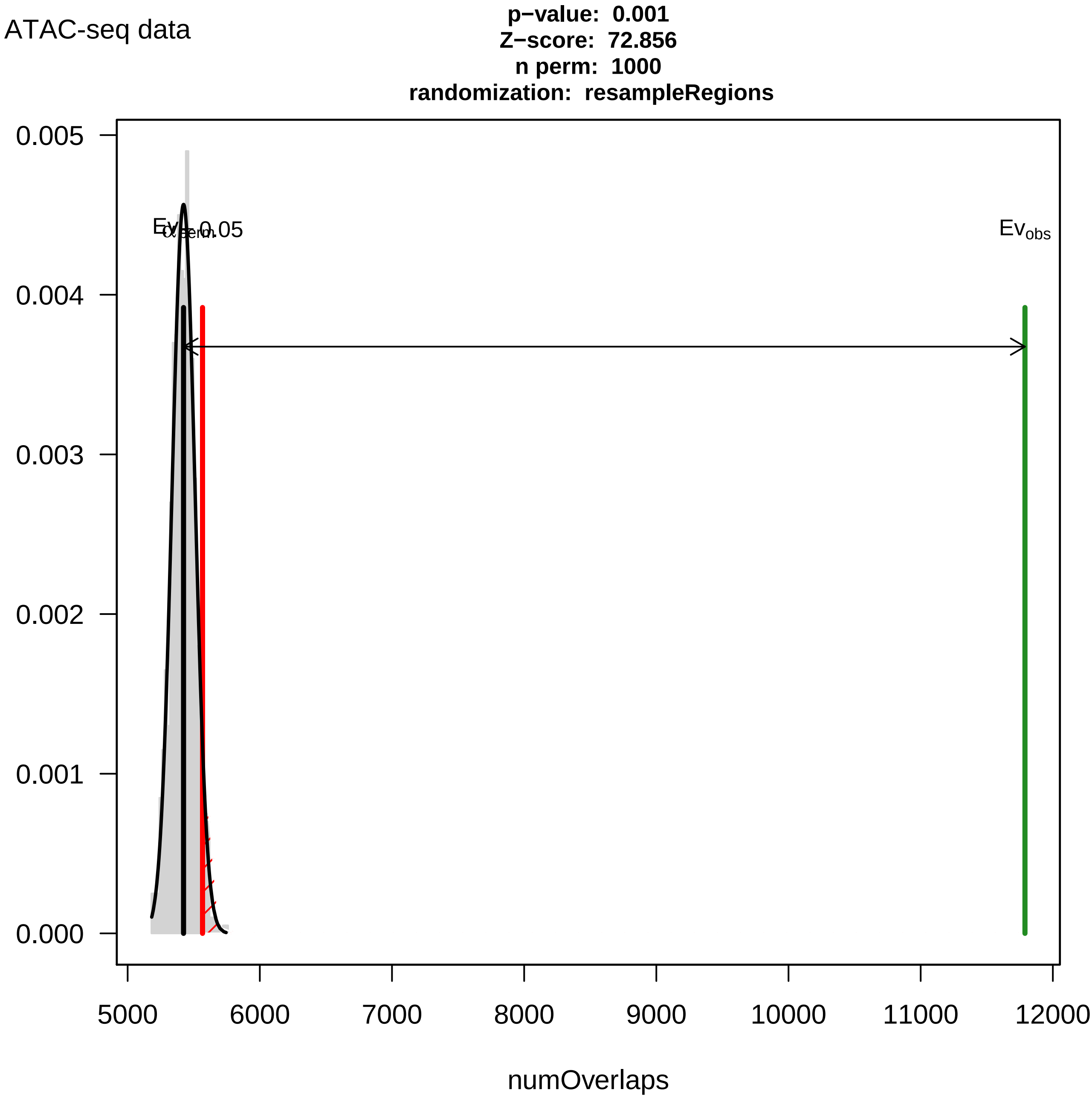

ATAC-seq data
p−value: 0.001
Z−score: 72.856
n perm: 1000
randomization: resampleRegions
0.005
0.004
0.003
0.002
0.001
0.000
Ev perm
α = 0.05
Ev obs
5000
6000
7000
8000
9000
10000
11000
12000
numOverlaps

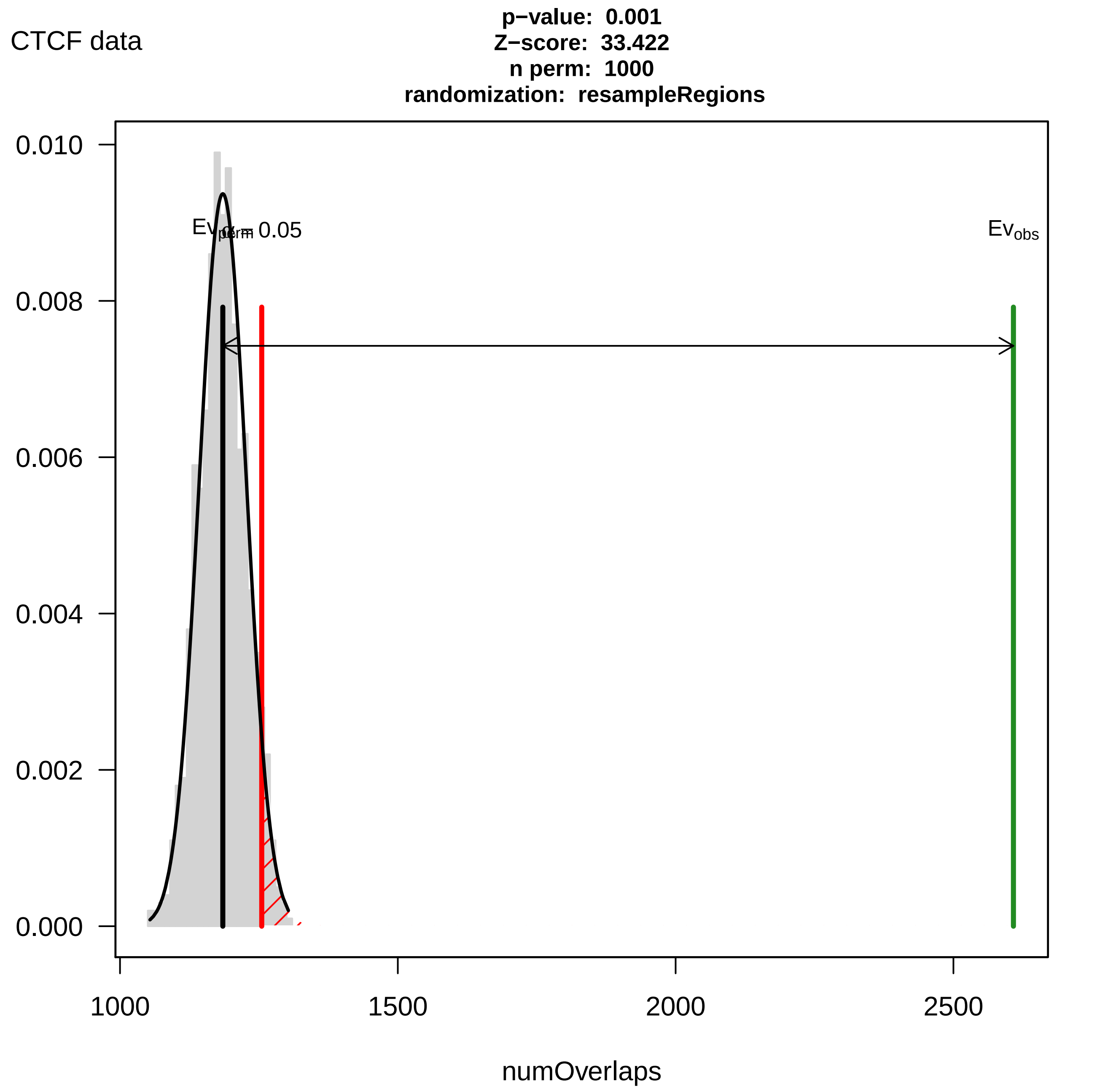

CTCF data
p−value: 0.001
Z−score: 33.422
n perm: 1000
randomization: resampleRegions
0.010
0.008
0.006
0.004
0.002
0.000
Ev perm
α = 0.05
Ev obs
1000
1500
2000
2500
numOverlaps

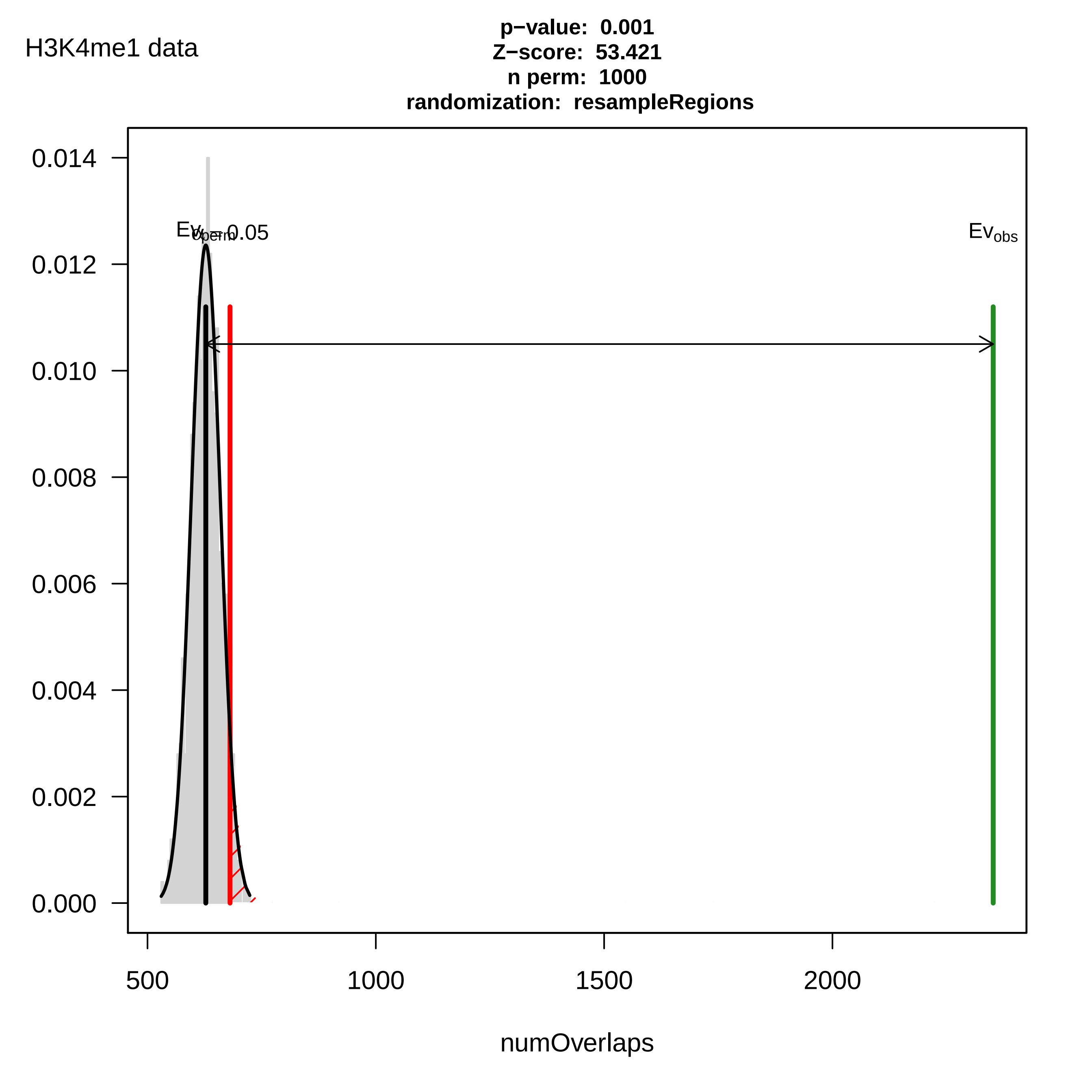
H3K4me1 data
p−value: 0.001
Z−score: 53.421
n perm: 1000
randomization: resampleRegions
0.014
0.012
0.010
0.008
0.006
0.004
0.002
0.000
Ev_{perm}
α = 0.05
Ev_{obs}
500
1000
1500
2000
numOverlaps

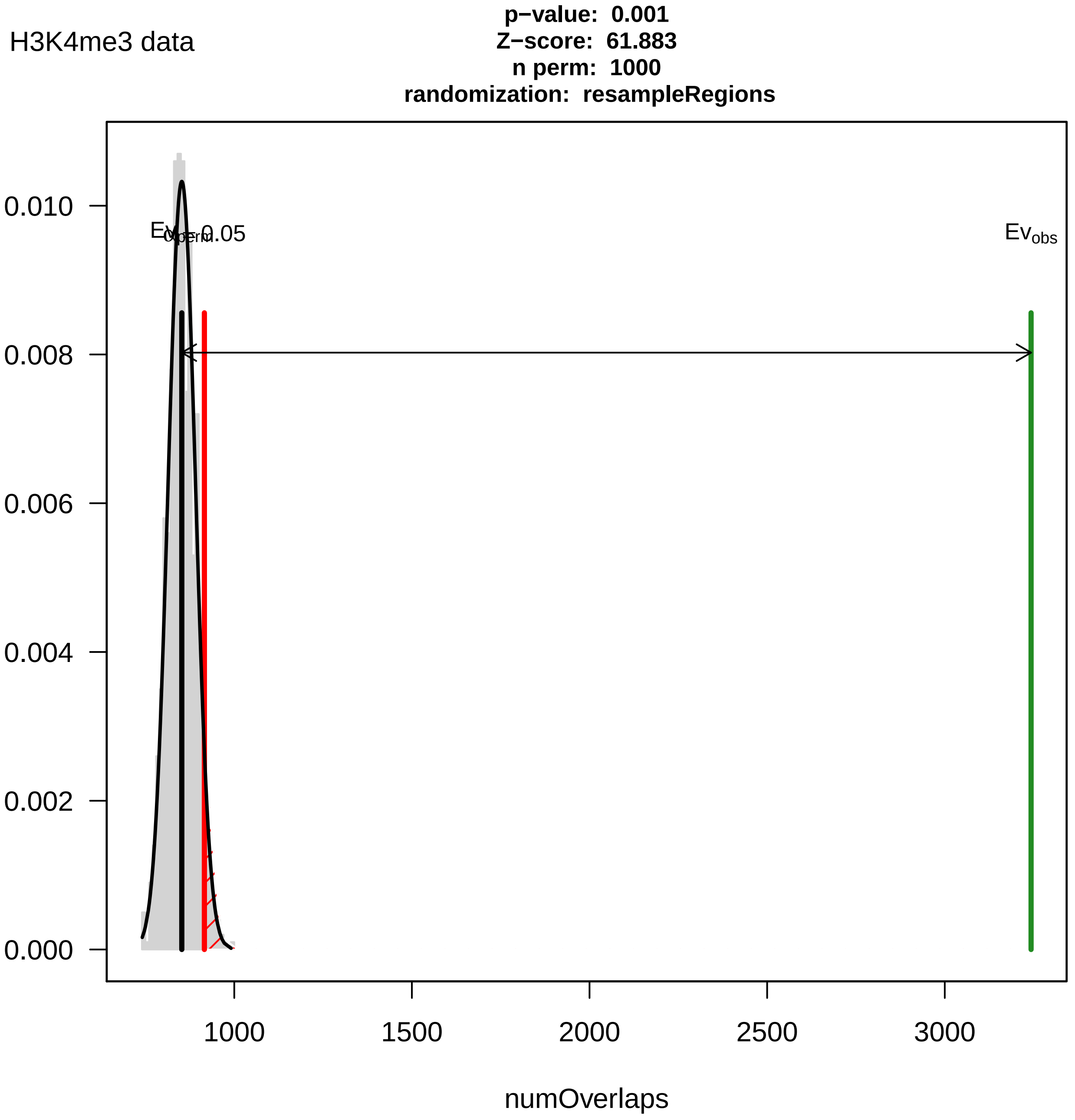
H3K4me3 data
p−value: 0.001
Z−score: 61.883
n perm: 1000
randomization: resampleRegions
Ev_perm
α = 0.05
Ev_obs
0.010
0.008
0.006
0.004
0.002
0.000
1000
1500
2000
2500
3000
numOverlaps

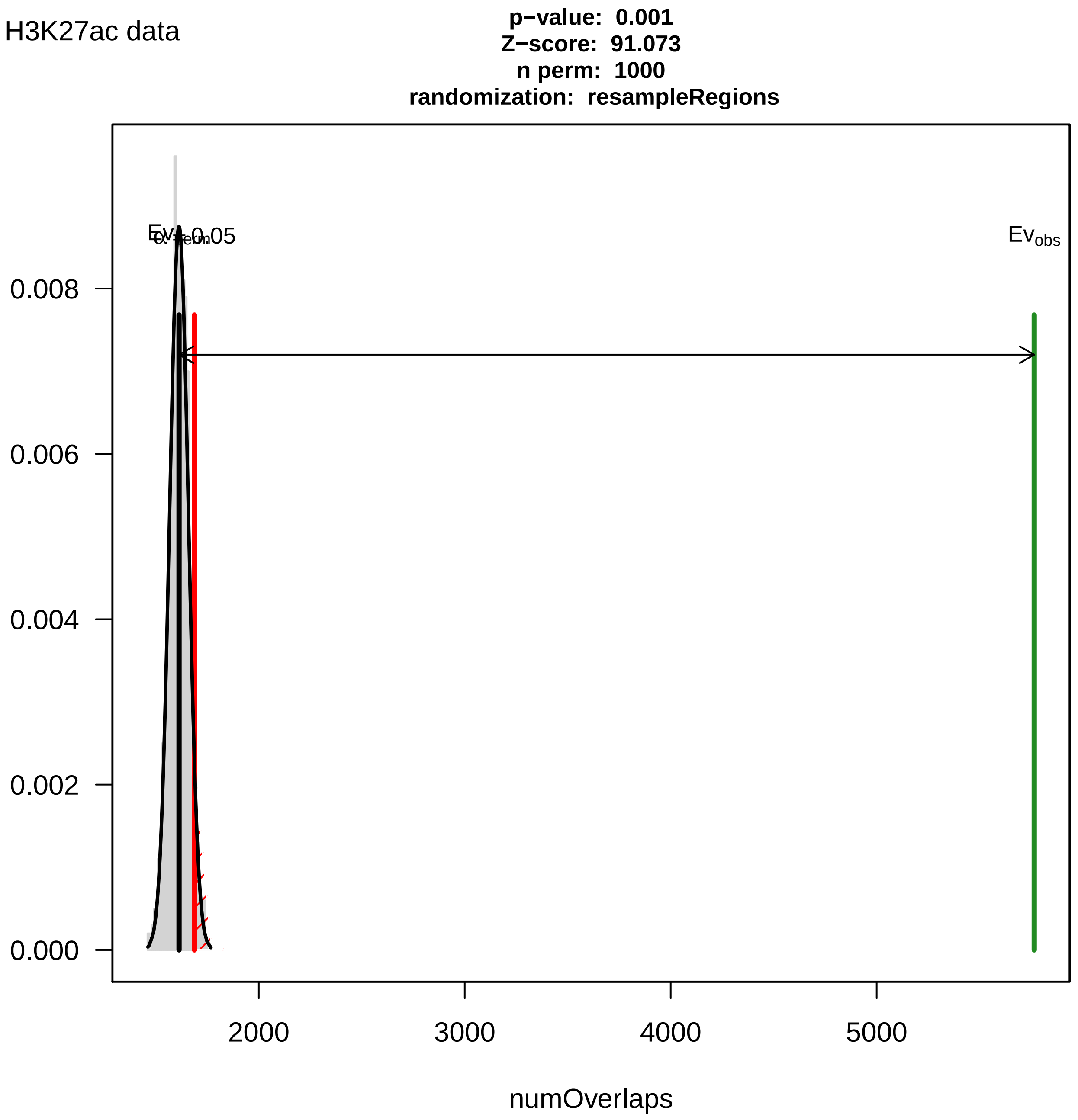
H3K27ac data
p−value: 0.001
Z−score: 91.073
n perm: 1000
randomization: resampleRegions
Ev_{perm}
$\alpha = 0.05$
Ev_{obs}
0.008
0.006
0.004
0.002
0.000
2000
3000
4000
5000
numOverlaps

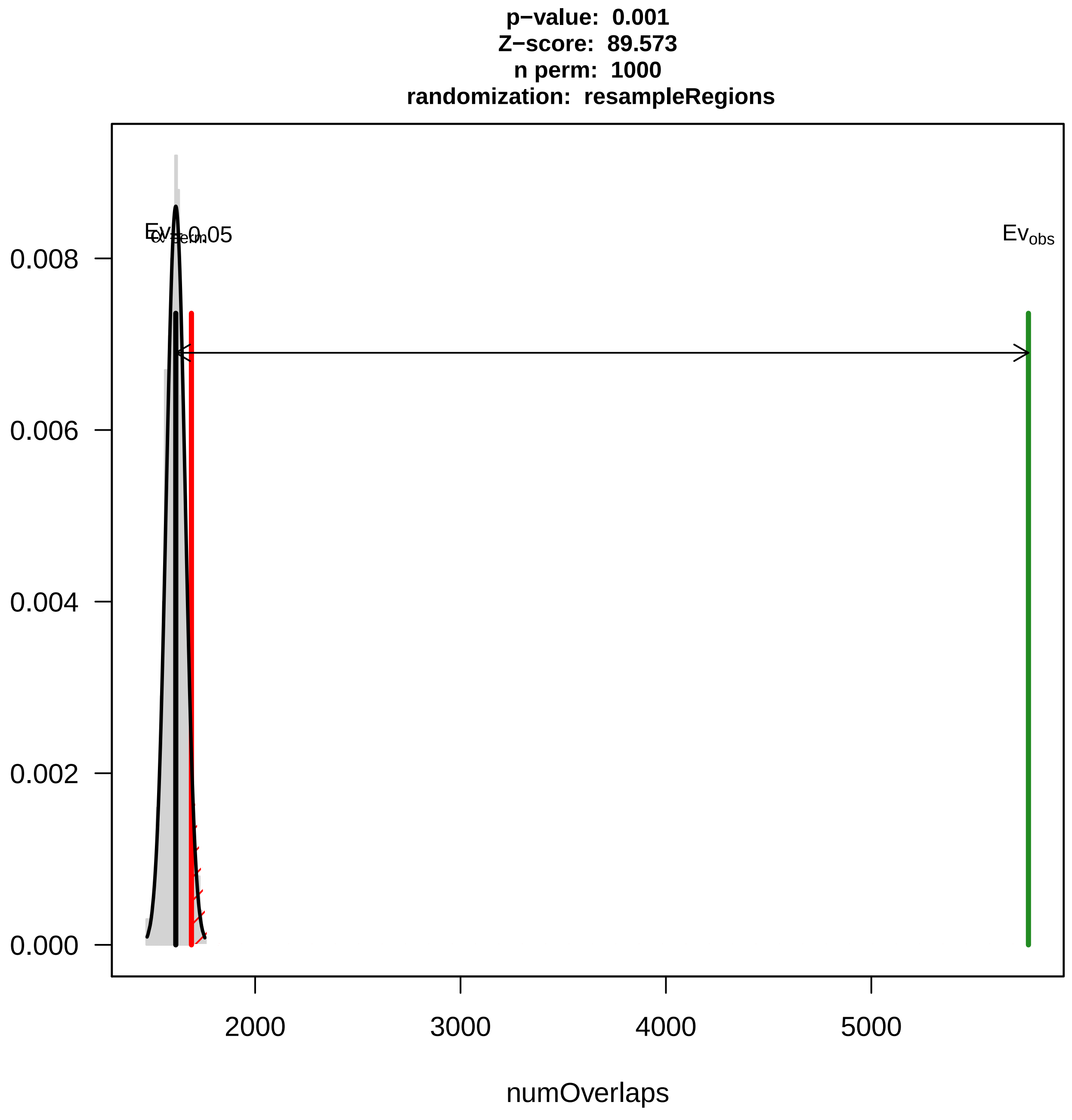
p−value: 0.001
Z−score: 89.573
n perm: 1000
randomization: resampleRegions
Ev_perm
α = 0.05
Ev_obs
0.000
0.002
0.004
0.006
0.008
2000
3000
4000
5000
numOverlaps

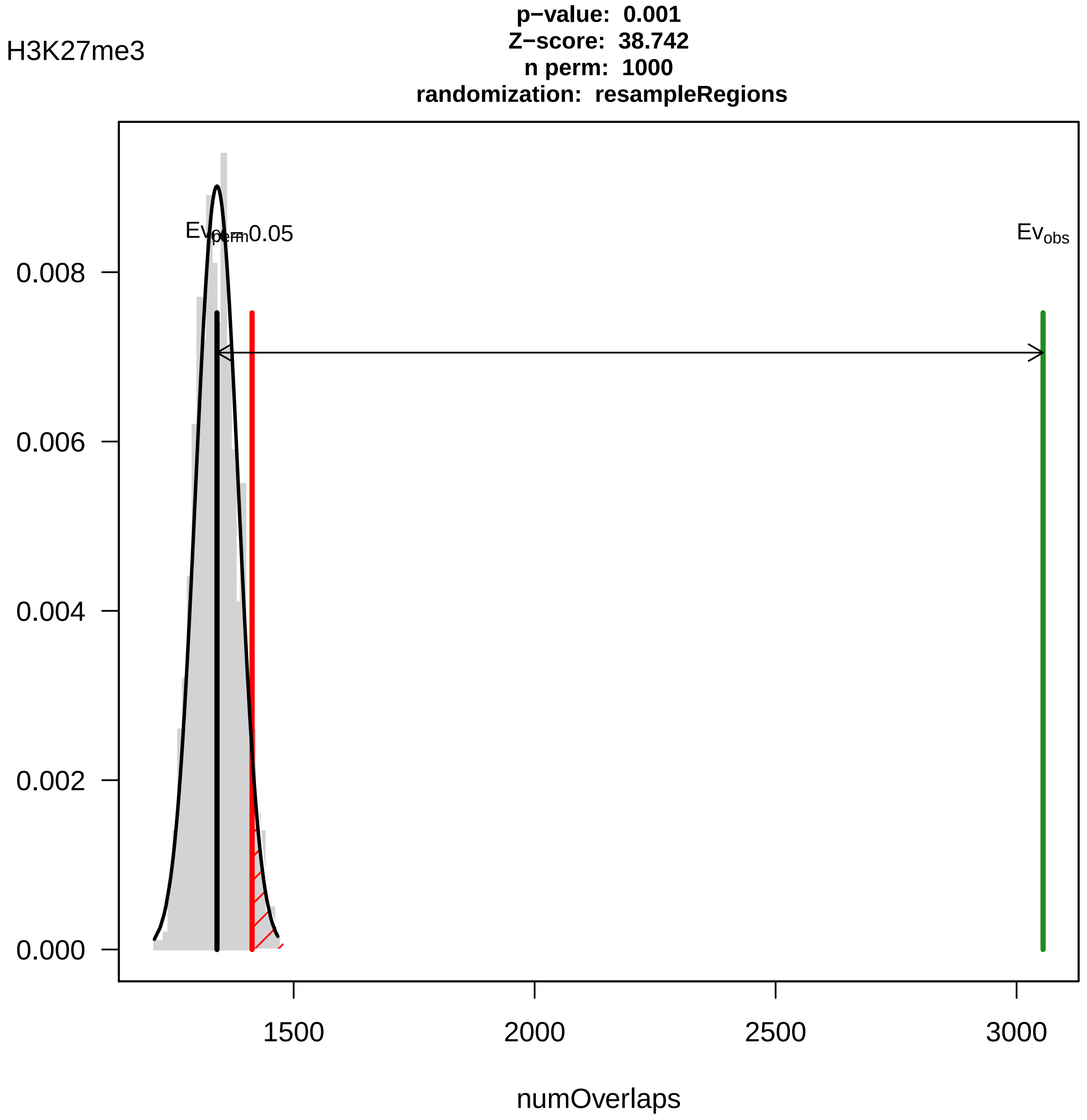

H3K27me3
p−value: 0.001
Z−score: 38.742
n perm: 1000
randomization: resampleRegions
Ev perm
α=0.05
Ev obs
0.000
0.002
0.004
0.006
0.008
1500
2000
2500
3000
numOverlaps
